# Anatomic reference measures for central airway anatomy in Indian adults: implications for precision airway management in surgical patient safety

**DOI:** 10.1186/s13037-025-00450-2

**Published:** 2025-10-01

**Authors:** Purnima Adhikari, Chandni Gupta, Koteshwara Prakashini, Rahul Magazine, Mohan K Manu, Santa Kumar Das, Sharma Paudel

**Affiliations:** 1 Department of Anatomy, Kasturba Medical College, Manipal, Manipal Academy of Higher Education, Manipal, Karnataka 576104 India; 2 Department of Radio Diagnosis and Imaging, Kasturba Medical College, Manipal, Manipal Academy of Higher Education, Manipal, Karnataka 576104 India; 3 Department of Respiratory Medicine, Kasturba Medical College, Manipal, Manipal Academy of Higher Education, Manipal, Karnataka 576104 India; 4 Department of Internal Medicine, Institute of Medicine, Tribhuvan University Teaching Hospital, Kathmandu, Nepal; 5 Department of Radio Diagnosis and Imaging, Institute of Medicine, Tribhuvan University Teaching Hospital, Kathmandu, Nepal

**Keywords:** Airway management, HRCT, Normative data, Precision medicine, Tracheobronchial anatomy

## Abstract

**Background:**

Despite the critical role of central airway dimensions in clinical practice, comprehensive normative data remain scarce globally, particularly for diverse ethnic populations. This study aims to establish the first high-resolution computed tomography (HRCT) based reference values for tracheobronchial anatomy in Indian adults, addressing a significant gap in precision medicine.

**Methods:**

This retrospective cross-sectional study was conducted at Kasturba Hospital, Manipal, India. HRCT chest scans performed between January 1, 2021, and March 31, 2024, were screened, and 503 adults (277 males, 226 females; aged 20–80 years) with normal findings were included. Primary outcomes were normative tracheal and bronchial dimensions (lengths, diameters, cross-sectional areas). Secondary outcomes included age and gender-based variations, correlations with demographics, and predictive models for airway device selection. Inclusion criteria were HRCT scans with normal thoracic findings and adequate inspiratory effort. Exclusion criteria included thoracic, pulmonary or cardiac abnormalities, prior airway surgery, presence of airway devices, or severe imaging artifacts. Data were analysed using t-test, one-way ANOVA, Pearson correlations, and multiple linear regression. Statistical significance was set at *p* < 0.05.

**Results:**

Indian males exhibited significantly larger airways than females (tracheal length: 109.5 ± 8.9 mm vs. 100.5 ± 7.4 mm, *p* < 0.001; distal tracheal area: 311.3 ± 111.1 mm² vs. 227.6 ± 92.9 mm², *p* < 0.001). Notably, 54.5% of subjects had a more vertical left bronchus, contradicting classical anatomical dogma. High rates of short right main bronchi (< 23 mm) were observed in 49.5% of subjects, increasing the risk of double-lumen endobronchial tube misplacement. The distal tracheal diameter was strongly correlated with bronchial dimensions (*r* = 0.621, *p* < 0.001), providing evidence-based device selection.

**Conclusion:**

This study provides the first population-specific normative data for central airway dimensions in Indian adults, revealing profound ethnic variations with immediate clinical implications for airway management, thoracic surgery, and personalised medical device design. The findings underscore the necessity of region-specific reference standards to optimise patient safety and highlight the influence of ethnicity on airway anatomy.

## Background

High-resolution computed tomography (HRCT) has emerged as the gold standard for non-invasive assessment of airway morphology, providing precise quantitative measurements of airway dimensions with excellent reproducibility [1]. This precision is paramount in surgical patient safety, particularly during lung isolation procedures, such as those employing a double-lumen endobronchial tube (DLT) or complex ventilatory strategies [2]. Computed tomography (CT) based assessments of the left mainstem bronchial diameter have proven especially useful for selecting the left-sided DLT (L-DLT) size, providing greater predictive accuracy, particularly for smaller tube sizes, than traditional methods based on tracheal diameter from chest radiographs [2, 3]. Beyond DLT, millimetre-level precision is crucial in procedures such as jet ventilation, where the catheter-to-airway diameter ratio must be carefully calibrated to balance oxygenation against barotrauma risk [4, 5]. Furthermore, improper tube sizing, such as the use of oversized endotracheal tubes (≥ 8 mm) in male patients, has been linked to an increased risk of posterior glottic stenosis, underscoring the need for population-specific airway sizing protocols to minimize iatrogenic complications [6].

Despite these significant advancements, robust data on normal adult central airway dimensions remain surprisingly limited. Existing morphometric studies often rely on cadaveric specimens, older imaging techniques with lower resolution, or are predominantly based on Western populations [7, 8]. This is particularly concerning given the well-established anatomical variability of the tracheobronchial tree across different racial and ethnic groups, highlighting the urgent need for more inclusive, population-specific reference standards [7, 9]. While paediatric airway dimensions have been extensively explored with CT, demonstrating correlations with age and height, comprehensive adult data for diverse populations remain essential, as paediatric data cannot be generalised to adults [10–12]. The absence of comprehensive adult data across diverse populations not only limits clinical decision-making but also poses a significant challenge for emerging personalised therapies, such as CT-based customizable tracheal implants for conditions like severe stenosis or tracheomalacia, where precise anatomical detail is critical for safe and effective treatment [13].

To address these significant gaps, this comprehensive cross-sectional study utilised HRCT imaging to establish normative reference values for central airway dimensions in the Indian adult population.

## Methods

This retrospective cross-sectional study aimed to establish normative reference values for tracheobronchial anatomy in Indian adults, hypothesising that central airway dimensions exhibit population-specific variability relevant to precision airway management. The study was conducted at Kasturba Hospital, Manipal, India, following approval from the Institutional Ethics Committee (IEC: 282/2024); individual consent was waived due to the retrospective design. Primary outcome measures were normative values of tracheal and bronchial dimensions (tracheal length, diameters, cross-sectional areas, and bronchial measurements) stratified by age and gender. The secondary outcome measures included assessing age and gender-based variations, correlation of airway dimensions with demographic parameters, and developing predictive models to guide device sizing and clinical airway management.

**Fig. 1:**
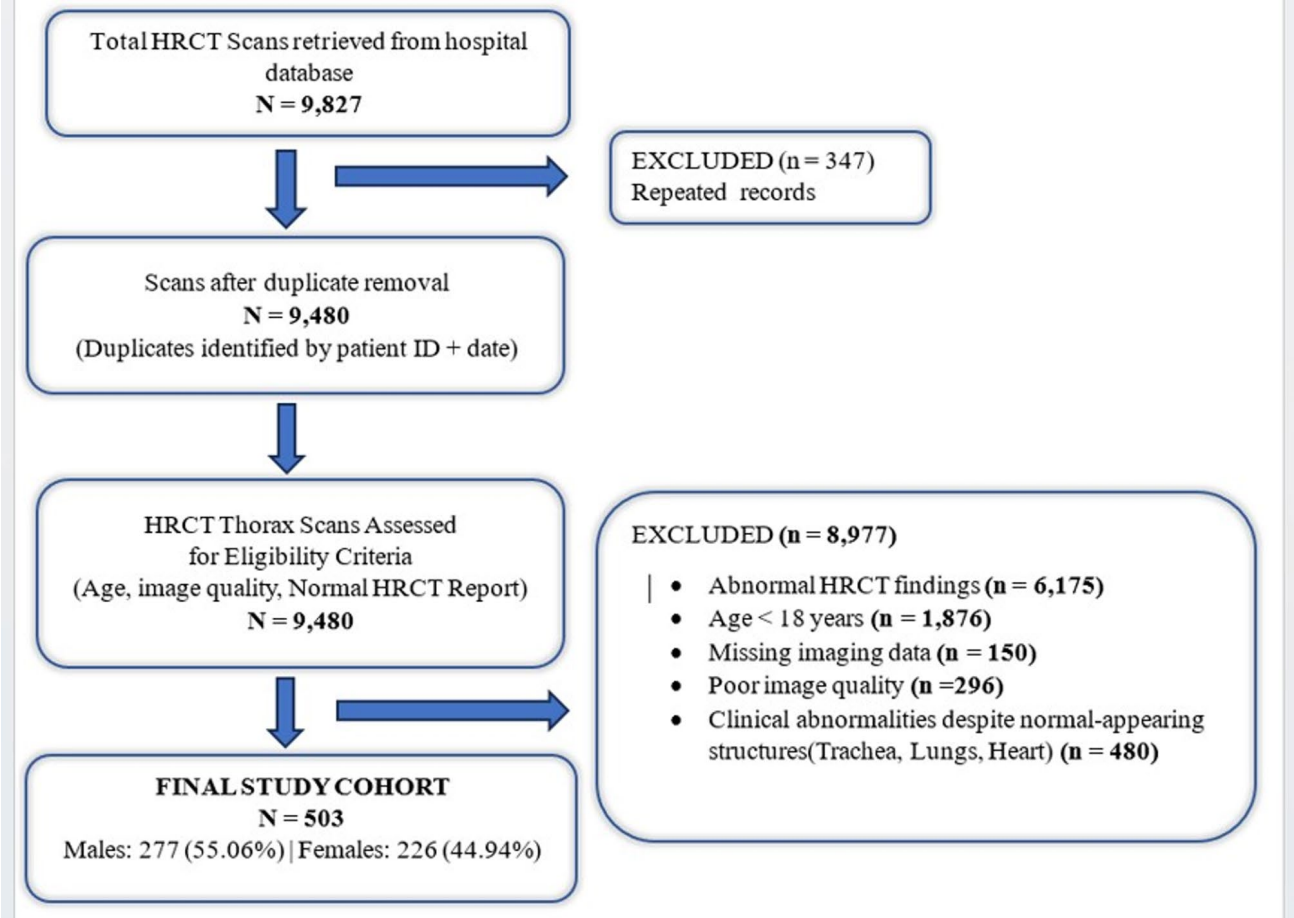
Flow chart for participant selection

A total of 9,827 HRCT thoracic scans, performed between January 1, 2021, and March 31, 2024, for various diagnostic indications, were screened. Of these, 503 participants (277 males, 226 females; age range: 20–80 years) were included based on predefined eligibility criteria. The details of participant selection are shown in Fig. 1. Inclusion criteria were adults with HRCT scans reported by radiologists as “no significant thoracic abnormalities” or “essentially normal study of the thorax” according to standard chest CT protocols; contiguous helical CT acquisition; image overlap, defined as slice thickness greater than the reconstruction increment; adequate inspiratory lung volume, indicated by a round tracheal shape and visible lung parenchyma between the heart and sternum; and absence of artifacts or only mild artifacts with minimal effect on central airway visualization. Exclusion criteria included thoracic cavity, pulmonary or cardiac abnormalities; prior tracheobronchial surgery or history of intubation; presence of airway devices; structural airway anomalies; poor inspiratory effort; and severe imaging artifacts.

All CT scans were performed using a Siemens Incisive 128 slice dual source CT scanner (Siemens Healthineers, Germany) with advanced dose optimisation including CARE Dose4D and Stellar detectors for enhanced image quality with collimation, 64 × 0.625 mm; pitch, 1.0 mm; digital matrix 512 × 512 pixels; tube potential, 120 kV and 0.35-second rotation time. Tube current was automatically adjusted using dose modulation. Acquisition time was 3.7 s. The scanned CT images were uploaded to the Picture Archiving and Communication System (PACS) and stored in DICOM format.

### Statistical analysis

Statistical analysis was performed using Jamovi statistical software (version 2.6.26, Sydney, Australia). Appropriate parametric tests were selected based on data distribution. Continuous variables were expressed as mean ± standard deviation and compared between groups using One-Way ANOVA followed by post-hoc Tukey’s test for multiple comparisons. Gender-based differences were assessed using independent t-tests. Pearson correlation coefficients were calculated to examine relationships between airway dimensions and demographic variables. Multiple linear regression models were constructed to identify predictors of bronchial dimensions, with model performance assessed using R² values and 95% confidence intervals. Statistical significance was set at *p* < 0.05, with Bonferroni correction applied for multiple comparisons where appropriate.

## Results

### Population characteristics

A total of 503 participants met the inclusion criteria, consisting of 277 males (55.06%) and 226 females (4.4.94%), with ages ranging from 20 to 80 years. The participants were divided into five age groups: young adults (Group 1: 20–30 years, *n* = 80; 24.69 ± 3.52 years), early middle-aged adults (Group 2: 31–40 years, *n* = 83; 35.93 ± 2.76 years), middle-aged adults (Group 3: 41–50 years, *n* = 99; 45.74 ± 2.81 years), late middle-aged adults (Group 4: 51–60 years, *n* = 115; 55.39 ± 2.97 years), and older adults (Group 5: 61–80 years, *n* = 126; 67.98 ± 5.71 years).

### Measurement

All CT scans were measured using Radiant DICOM Viewer 2024.2 (Medixant, Poznan, Poland). Measurements were performed on lung window images with a slice thickness of 0.625 mm.

**Fig. 2:**
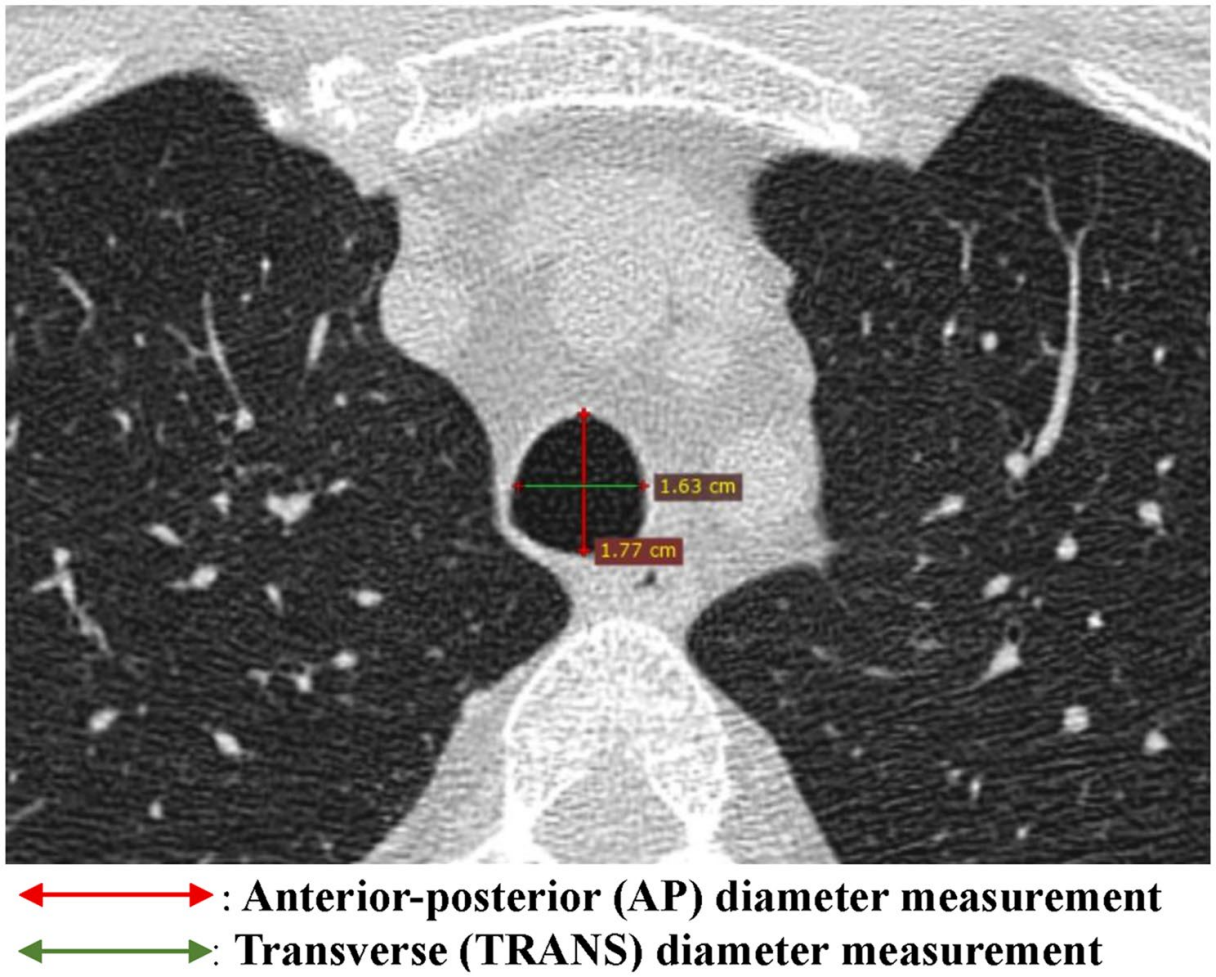
Axial chest CT reconstruction (lung window) demonstrating tracheal diameter measurements

The internal diameter of the trachea was first measured from the axial images (Fig. 2). Maximal tracheal diameters in the transverse plane, which included both anterior-posterior (AP) and transverse (TRANS) diameters along with cross-sectional area (CSA), were measured at four levels:

Level 1 (M1): Sub-cricoid Region

Anatomical landmark: Immediately inferior to the cricoid cartilage

- AP1, TRANS1, CSA1.

Level 2 (M2): Thoracic Inlet

Anatomical landmark: Clavicular level

- AP2, TRANS2, CSA2.

Level 3 (M3): Mid-thoracic Trachea

Anatomical landmark: Superior border of the aortic arch

- AP3, TRANS3, CSA3.

Level 4 (M4): Distal Trachea

Anatomical landmark: 1 cm proximal to the carina

- AP4, TRANS4, CSA4.

Subsequently, multiplanar reconstruction (MPR) was performed along the ordinate axes of the left and right bronchus at the carina plane of the axial images obtained through each CT scan. Total tracheal length (TL) was measured as the distance between the lower border of the cricoid cartilage and the carina. Further, the intrathoracic length of the trachea (I-TL) was measured from the thorax inlet, defined as the first image slice in which both lung apices became visible, to the carina. The cervical length of the trachea (C-TL) was measured from the cricoid cartilage to the thoracic inlet. Additionally, the ratio between the thoracic and cervical tracheal length was calculated for each subject.

The length of the right main stem bronchus (RBL) and the length of the left main stem bronchus (LBL) were measured as the distances between the tracheal bifurcation point and the point where RBL or LBL divides into the secondary bronchi, respectively. The measurements were taken on the coronal plane in the axis of the RBL and LBL, on the frame that showed a clear tracheal carina and the largest diameter of the respective main stem bronchus.

We used three methods to approximate the length of the right main stem bronchus [14].

**Fig. 3:**
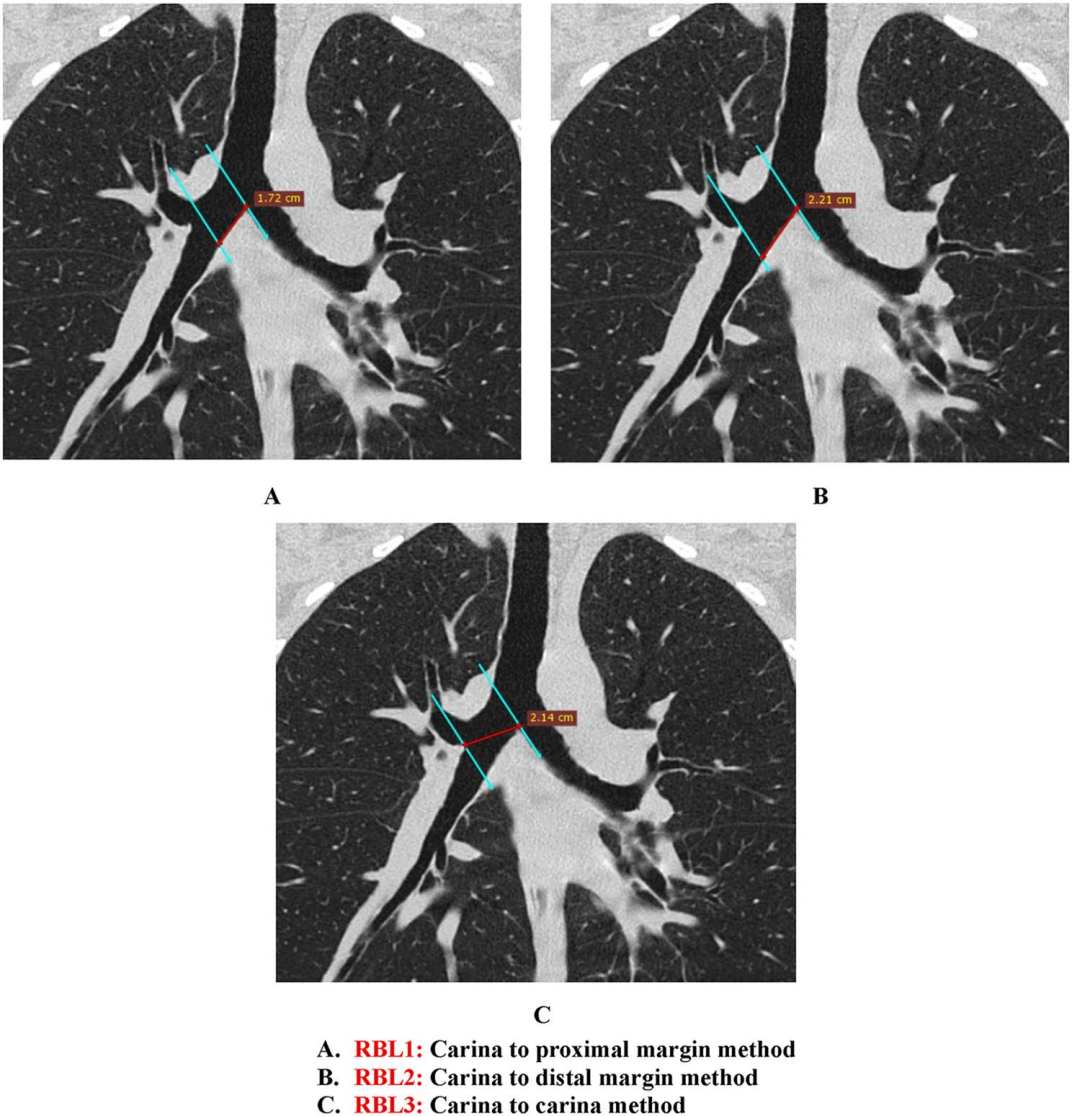
Coronal chest CT reconstruction (lung window) demonstrating right main stem bronchus length measurements using three methods. **A**. RBL1: Carina to proximal margin method. **B**. RBL2: Carina to distal margin method. **C**. RBL3: Carina to carina method

1. RBL1: Carina-proximal margin method (distance between the tracheal carina and the proximal margin of the right upper lobe orifice**)** (Fig. 3A).
2. RBL2: Carina-distal margin method (distance between the tracheal carina and the distal margin of the right upper lobe orifice) (Fig. 3B).
3. RBL3: Carina-carina method (distance between the tracheal carina and the first right interlobar carina of the right upper lobe orifice) (Fig. 3C).

**Fig. 4:**
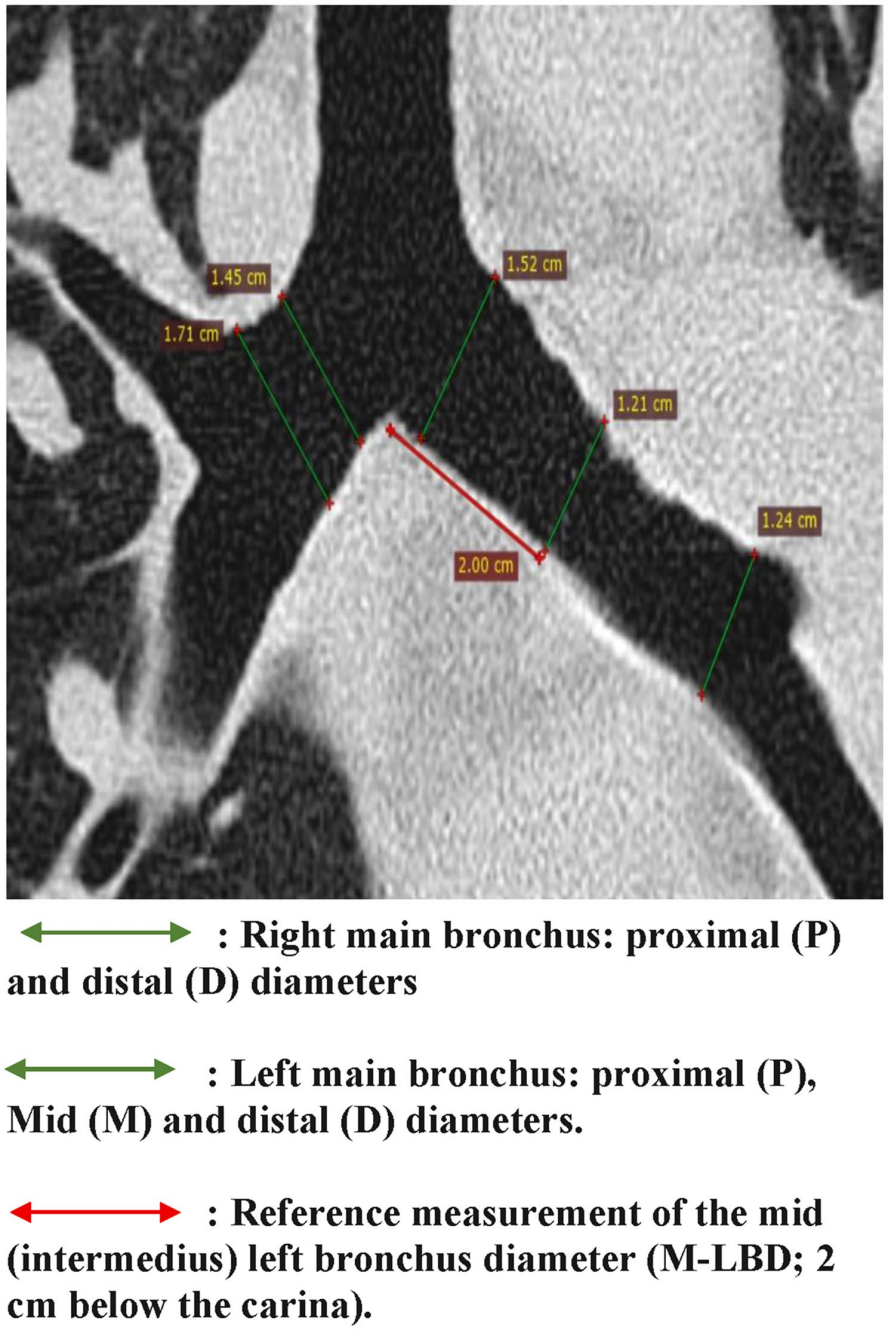
Coronal chest CT reconstruction (lung window) demonstrating main bronchus diameter measurements

The RBL and LBL were divided into several sections. For each subject, the length of the RBL was divided into two equal sections: proximal and distal, and the LBL was divided into three equal sections: proximal, middle, and distal. The internal diameters were measured perpendicular to their long axes on MPR images (Fig. 4): at the proximal (P-RBD) and distal (D-RBD) ends of the right main stem bronchus, and at the proximal (P-LBD), mid (M-LBD; intermedius, 2 cm below carina) and distal (D-LBD) ends of the left main stem bronchus, using the same method as for the right upper lobe bronchus orifice.

**Fig. 5:**
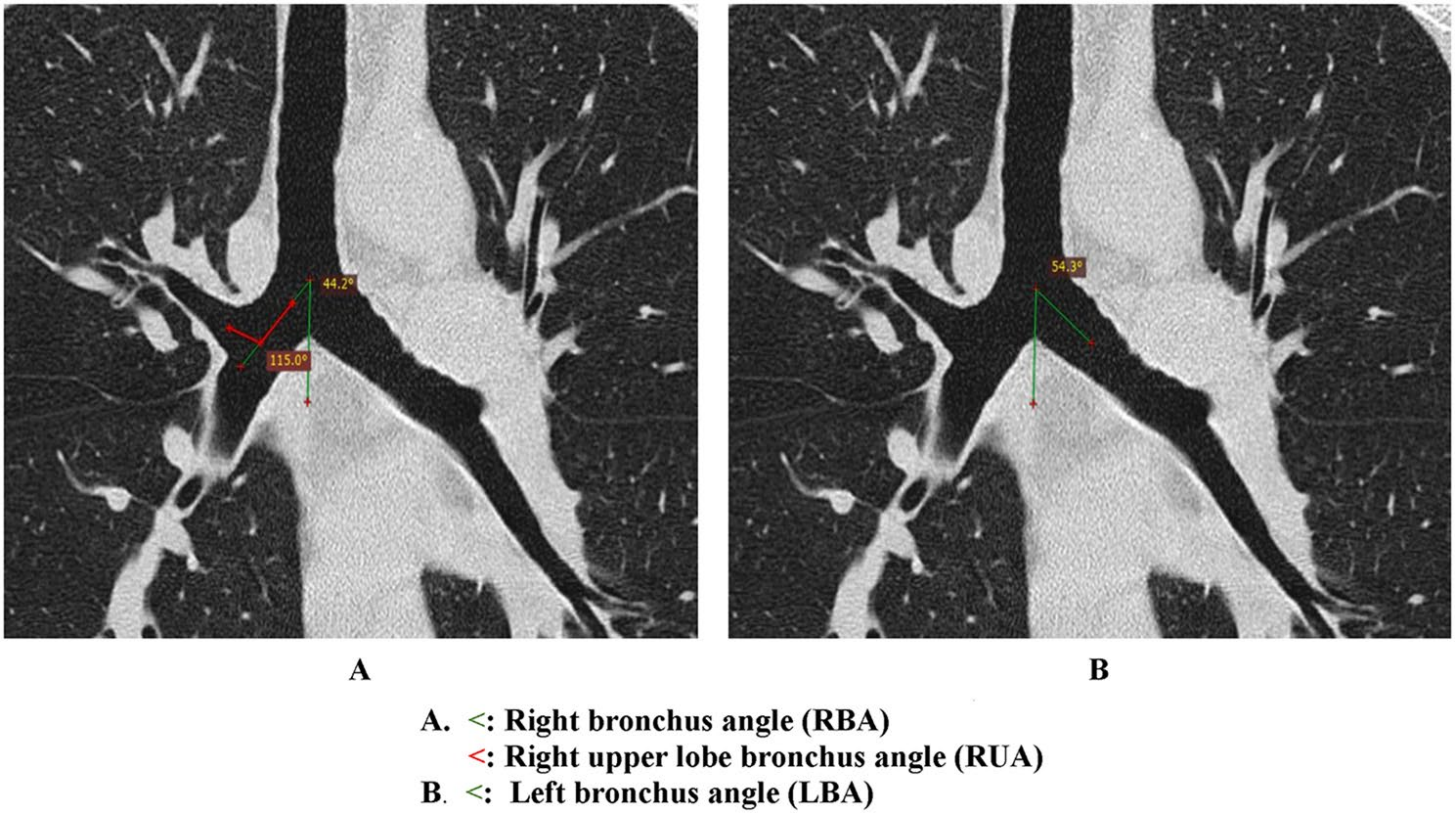
Coronal chest CT reconstructions (lung window) demonstrating bronchial angle measurements

The right bronchus angle(RBA) was measured as the angle between the vertical line passing through the lower point of the tracheal bifurcation and the line drawn on the mid-axis of the RBL (Fig. 5A). The left bronchus angle (LBA) was measured as the angle between the vertical line passing through the lower point of the tracheal bifurcation and the line drawn on the mid-axis of the LBL (Fig. 5B). The angle at the intersection of the lower borders of the right and left main bronchus was measured as the subcarinal angle (SCA). The angulation of the right upper lobe bronchus was measured as the angle between the vertical line passing through the central axis of the right upper lobe bronchus and the vertical line passing through the mid-axis of the right main stem bronchus.

Reference ranges (5th–95th percentiles) for central airway dimensions were established (Table 1). Tracheal length ranged from 95.9 to 124.0 mm in men and 90.0–111.0 mm in women. Distal tracheal cross-sectional area showed marked variability, measuring 145–519 mm² in men and 108–375 mm² in women. Bronchial angles were broadly comparable between genders, although subcarinal angles demonstrated the widest dispersion (42.8–105° in men; 48.3–110° in women). Bronchial dimensions consistently exhibited larger values in men, including proximal right bronchial diameter (11.2–20.1 mm vs. 10.2–17.3 mm) and proximal left bronchial diameter (9.66–18.8 mm vs. 8.83–15.7 mm).

**Table 1:** Gender-Specific central airway reference values

Parameter	Male		Female	
	5th percentile	95th percentile	5th percentile	95th percentile
Tracheal Length Measurements				
TL (mm)	95.9	124	90.0	111
I-TL (mm)	59.5	93.3	52.1	80.8
C-TL (mm)	18.2	48.2	20.4	49.0
I-TL /C-TL (mm)	1.34	4.97	1.14	3.67
Tracheal Cross-Sectional Dimensions				
AP1 (mm)	14.2	21.5	12.3	18.8
TRANS1 (mm)	12.1	19.4	11.3	17.7
CSA1 (mm²)	115	297	100.0	245
AP2 (mm)	12.6	22.9	9.62	18.0
TRANS2 (mm)	12.7	20.5	11.5	17.9
CSA2 (mm²)	126	331	104	252
AP3 (mm)	12.1	20.7	9.83	17.8
TRANS3 (mm)	13.0	21.1	11.4	17.9
CSA3 (mm²)	132	351	103	252
AP4 (mm)	11.5	19.0	8.92	16.6
TRANS4 (mm)	13.6	25.7	11.7	21.8
CSA4 (mm²)	145	519	108	375
Bronchial Angle Measurements				
RBA (°)	26.2	51.2	27.3	53.4
LBA (°)	27.4	50.2	29.0	50.2
SCA (°)	42.8	105	48.3	110
RUA (°)	100	131	100	136
Right Bronchial Measurements				
RBL1 (mm)	8.32	25.5	7.33	21.9
RBL2 (mm)	16.9	31.6	14.4	28.6
RBL3 (mm)	19.6	32.7	18.4	30.1
P-RBD (mm)	11.2	20.1	10.2	17.3
D-RBD (mm)	11.8	20.6	10.0	17.9
RUBD (mm)	5.81	13.8	5.63	12.3
Left Bronchial Measurements				
LBL (mm)	38.2	56.1	34.5	51.0
P-LBD (mm)	9.66	18.8	8.83	15.7
M-LBD (mm)	8.14	14.1	6.90	12.4
D-LBD (mm)	6.73	16.8	5.56	15.1

TL – Total tracheal length; I-TL – Intrathoracic tracheal length; C-TL – Cervical tracheal length; I-TL/C-TL – Ratio of intrathoracic to cervical tracheal length; AP1–AP4 – Anteroposterior tracheal diameters at levels 1 to 4 (sub-cricoid, thoracic inlet, mid-thoracic, distal trachea); TRANS1–TRANS4 – Transverse tracheal diameters at levels 1 to 4; CSA1–CSA4 – Cross-sectional areas of the trachea at levels 1 to 4; RBA – Right bronchial angle; LBA – Left bronchial angle; SCA – Subcarinal angle; RUA – Right upper lobe bronchial angle; RBL1 – Right bronchus length (carina to proximal margin of right upper lobe orifice); RBL2 – Right bronchus length (carina to distal margin of right upper lobe orifice); RBL3 – Right bronchus length (carina to interlobar carina of right upper lobe orifice); P-RBD – Proximal right bronchus diameter; D-RBD – Distal right bronchus diameter; RUBD – Right upper bronchus diameter; LBL – Left bronchus length; P-LBD – Proximal left bronchus diameter; M-LBD – Mid (intermedius) left bronchus diameter (2 cm below carina); D-LBD – Distal left bronchus diameter

### Gender based comparison of central airway dimensions

Central airway measurements showed significant gender-based differences, with males having larger dimensions than females across most parameters (*p* < 0.05) (Table 2).

Males exhibited longer tracheal length than females (109.5 ± 8.9 mm vs. 100.5 ± 7.4 mm, *p* < 0.001), driven by intrathoracic segment differences (75.5 ± 10.3 mm vs. 66.0 ± 8.8 mm, *p* < 0.001). Cervical lengths were similar (*p* = 0.494), resulting in higher thoracic/cervical ratios in males (2.5 ± 1.3 vs. 2.1 ± 0.8, *p* < 0.001).

Tracheal dimensions were consistently larger in males throughout all measured levels, most notably at the distal trachea (CSA4: 311.3 ± 111.1 vs. 227.6 ± 92.9 mm², *p* < 0.001). Bronchial measurements showed parallel trends, with males exhibiting longer bronchial lengths and wider diameters (*p* < 0.005). For angles, females had slightly but significantly larger left bronchial (40.1 ± 6.8 vs. 38.4 ± 7.1) and subcarinal angles (76.8 ± 18.1 vs. 72.1 ± 19.6; both *p* = 0.005), while right-sided angles, including the right bronchial and right upper lobe angles, showed no gender-based significant differences (*p* > 0.05).

**Table 2:** Central airway dimensions by gender

Parameter	Male (*n* = 277)	Female (*n* = 226)	Total (*n* = 503)	*p* value
Tracheal Length				
TL (mm)	109.5 ± 8.9	100.5 ± 7.4	105.4 ± 9.4	< 0.001
I-TL (mm)	75.5 ± 10.3	66.0 ± 8.8	71.3 ± 10.8	< 0.001
C-TL (mm)	33.9 ± 9.5	34.5 ± 8.5	34.2 ± 9.1	0.494
I-TL/ C-TL (mm)	2.5 ± 1.3	2.1 ± 0.8	2.3 ± 1.1	< 0.001
Tracheal Cross-Sectional Dimensions				
AP1 (mm)	17.5 ± 2.2	16.1 ± 2.0	16.9 ± 2.2	< 0.001
TRANS1 (mm)	16.0 ± 2.1	15.4 ± 1.9	15.7 ± 2.1	0.001
CSA1 (mm²)	204.8 ± 53.6	188.5 ± 44.3	197.5 ± 50.3	< 0.001
AP2 (mm)	17.9 ± 3.2	13.5 ± 2.6	15.9 ± 3.6	< 0.001
TRANS2 (mm)	16.6 ± 2.5	14.7 ± 2.2	15.7 ± 2.6	< 0.001
CSA2 (mm²)	221.8 ± 66.3	173.3 ± 54.0	199.9 ± 65.6	< 0.001
AP3 (mm)	16.7 ± 2.7	13.7 ± 2.4	15.4 ± 3.0	< 0.001
TRANS3 (mm)	17.0 ± 2.5	14.6 ± 2.0	15.9 ± 2.6	< 0.001
CSA3 (mm²)	231.8 ± 67.7	171.3 ± 47.1	204.6 ± 66.5	< 0.001
AP4 (mm)	15.7 ± 2.4	12.9 ± 2.2	14.4 ± 2.7	< 0.001
TRANS4 (mm)	19.6 ± 3.6	16.7 ± 3.3	18.3 ± 3.7	< 0.001
CSA4 (mm²)	311.3 ± 111.1	227.6 ± 92.9	273.6 ± 111.3	< 0.001
Bronchial Angles				
RBA (°)	39.1 ± 7.5	39.1 ± 7.6	39.1 ± 7.5	0.996
LBA (°)	38.4 ± 7.1	40.1 ± 6.8	39.1 ± 7.0	0.005
SCA (°)	72.1 ± 19.6	76.8 ± 18.1	74.2 ± 19.1	0.005
RUA(°)	115.8 ± 10.2	116.5 ± 11.4	116.1 ± 10.8	0.464
Right Bronchial Measurements				
RBL1 (mm)	16.2 ± 5.3	15.0 ± 4.5	15.7 ± 5.0	0.005
RBL2 (mm)	23.9 ± 4.4	22.2 ± 4.2	23.1 ± 4.4	< 0.001
RBL3 (mm)	25.8 ± 4.0	24.0 ± 3.7	25.0 ± 4.0	< 0.001
Pe-RBD(mm)	15.4 ± 2.8	13.4 ± 2.3	14.5 ± 2.8	< 0.001
D-RBD (mm)	15.8 ± 2.7	13.8 ± 2.4	14.9 ± 2.8	< 0.001
RUBD (mm)	9.7 ± 2.5	8.9 ± 2.2	9.3 ± 2.4	< 0.001
Left Bronchial Measurements				
LBL(mm)	46.9 ± 5.5	43.2 ± 5.1	45.2 ± 5.6	< 0.001
P-LBD(mm)	13.9 ± 2.9	12.2 ± 2.2	13.1 ± 2.7	< 0.001
M-LBD (mm)	11.2 ± 1.9	9.8 ± 1.8	10.6 ± 2.0	< 0.001
D-LBD (mm)	11.5 ± 3.0	9.6 ± 2.8	10.6 ± 3.0	<0.001

Data are presented as mean ± standard deviation. Comparisons were performed using an independent t-test.*Statistically significant (*p* < 0.05)

**Table 3a:** Tracheal dimensions by age group

Parameters	Group 1 (*n* = 80)	Group 2 (*n* = 83)	Group 3 (*n* = 99)	Group 4 (*n* = 115)	Group 5 (*n* = 126)	*p* value
Age	24.69 ± 3.52	35.93 ± 2.76	45.74 ± 2.81	55.39 ± 2.97	67.98 ± 5.71	< 0.001
TL	103.72 ± 9.30	104.11 ± 9.21	105.95 ± 10.30	105.55 ± 8.33	106.87 ± 9.60	0.118
I-TL	71.05 ± 11.14	71.66 ± 11.80	69.37 ± 11.07	71.03 ± 9.42	72.80 ± 10.59	0.227
C-TL	32.67 ± 9.12	32.45 ± 8.92	36.58 ± 8.49	34.52 ± 8.75	34.07 ± 9.51	0.011
I-TL/C-TL	2.51 ± 1.54	2.52 ± 1.30	2.06 ± 0.77	2.26 ± 0.92	2.41 ± 1.11	0.008
AP1	16.55 ± 1.94	16.98 ± 2.19	16.84 ± 2.19	16.99 ± 1.92	17.01 ± 2.64	0.530
TRANS1	15.33 ± 1.90	15.82 ± 2.15	15.59 ± 2.00	15.81 ± 1.83	15.92 ± 2.31	0.280
CSA1	187.45 ± 45.33	200.29 ± 53.70	194.16 ± 47.36	198.92 ± 43.86	203.39 ± 57.50	0.207
AP2	15.29 ± 3.12	15.61 ± 3.62	16.16 ± 3.46	15.81 ± 3.64	16.30 ± 4.07	0.264
TRANS2	15.18 ± 2.02	15.82 ± 2.24	15.72 ± 2.53	15.74 ± 2.84	16.09 ± 2.78	0.095
CSA2	184.27 ± 48.24	200.63 ± 56.53	199.05 ± 63.07	200.89 ± 75.86	209.56 ± 71.22	0.042
AP3	14.84 ± 2.50	15.45 ± 2.93	15.61 ± 2.99	15.43 ± 3.00	15.38 ± 3.34	0.390
TRANS3	14.98 ± 2.06	15.69 ± 2.48	15.66 ± 2.40	16.25 ± 2.62	16.60 ± 2.82	< 0.001
CSA3	179.64 ± 47.96	198.18 ± 61.93	197.05 ± 59.99	212.85 ± 68.92	222.71 ± 75.71	< 0.001
AP4	13.84 ± 2.15	14.32 ± 2.61	14.63 ± 2.84	14.59 ± 2.65	14.64 ± 2.99	0.115
TRANS4	17.67 ± 3.45	18.60 ± 4.05	17.86 ± 3.54	18.53 ± 3.77	18.58 ± 3.73	0.230
CSA4	254.67 ± 97.96	284.57 ± 125.92	260.23 ± 101.58	280.80 ± 112.39	282.09 ± 114.06	0.182

### Age-related changes in central airway dimensions

While total tracheal length remained consistent across age groups (*p* = 0.118), significant variation was observed in cervical tracheal length (*p* = 0.011), with post-hoc Tukey tests indicating increased cervical segments in middle-aged adults relative to younger ones (Group:1 < 3, *p* = 0.033; Group:2 < 3, *p* = 0.018). Progressive changes were noted in tracheal dimensions, especially at the mid-thoracic level, where the transverse diameter and cross-sectional area increased significantly with age (*p* < 0.001). Multiple comparisons demonstrated significant enlargement between the younger and older age groups (Group:1 < 4, *p* = 0.004; Group: 1 < 5, *p* < 0.001; Group: 3 < 5, *p* = 0.028) (Table 3A).

Parameters for bronchial angulation (Table 3B) demonstrated significant age-associated widening of right bronchial angle (*p* = 0.012) (Group:1 < 2, *p* = 0.046; Group:1 < 5, *p* = 0.042) and subcarinal angle (*p* = 0.005) (Group:1 < 2, *p* = 0.006; Group:1 < 4, *p* = 0.035) with older cohorts maintaining consistently greater angles. Most of the bronchial dimensions exhibited no notable change, except for the mid left bronchus diameter, which showed a significant increase in diameter with age (Group: 1 < 5, *p* = 0.006) (Table 3C).

### Correlation analysis

The dimensions of the airway were poorly correlated with age. Age demonstrated the strongest associations, particularly at the mid thoracic trachea with TRANS3 (*r* = 0.223, *p* < 0.001) and AREA3 (*r* = 0.231, *p* < 0.001). M-LBD exhibited a significant but weak age correlation (*r* = 0.173, *p* < 0.001). Total tracheal length correlated weakly with age (*r* = 0.098, *p* = 0.029), while individual thoracic and cervical segments showed no age-related associations.

Strong anatomical correlations were observed between airway measurements. Total tracheal length correlated strongly with thoracic length (*r* = 0.603, *p* < 0.001) and moderately with cervical length (*r* = 0.321, *p* < 0.001). A strong inverse relationship was observed between cervical tracheal length and thoracic-to-cervical ratio (*r* = -0.845, *p* < 0.001). Transverse diameters and cross-sectional areas demonstrated near-perfect correlations at all levels (*r* > 0.99, *p* < 0.001). Adjacent anteroposterior measurements showed strong correlations: AP2-AP3 (*r* = 0.816, *p* < 0.001) and AP3-AP4 (*r* = 0.813, *p* < 0.001). Strong correlations were observed between distal tracheal transverse diameter and proximal bronchial diameters: left bronchus (*r* = 0.605, *p* < 0.001) and right bronchus (*r* = 0.621, *p* < 0.001).

### Predictive modelling for clinical application

#### Regression equations

Multiple linear regression analysis identified TRANS4 as the strongest predictor for bronchial dimensions (Table 4).

#### P-LBD models

**Crude Model: P-LBD = 5.047 + 0.442 × TRANS4**

- R² = 0.367, F(1,501) = 290.0, *p* < 0.001

**Adjusted Model: P-LBD = 4.885 + 0.414 × TRANS4 + 0.008 × Age + 0.490 × Gender**

- R² = 0.376, F(3,499) = 100.0, *p* < 0.001

#### P-RBD models

**Crude Model: P-RBD = 6.036 + 0.465 × TRANS4**

- R² = 0.386, F(1,501) = 315.0, *p* < 0.001

**Adjusted Model: P-RBD = 6.043 + 0.422 × TRANS4 + 0.007 × Age + 0.786 × Gender**

- R² = 0.405, F(3,499) = 113.0, *p* < 0.001

TRANS4 shows consistent positive associations with both proximal bronchus diameter outcomes, with P-RBD demonstrating slightly stronger associations than P-LBD.

## Discussion

### Tracheal length

Our data underscore significant anatomical variability in human tracheal length. Notably, 23.45% of patients (17.29% females and 6.16% males) had a tracheal length shorter than 10 cm. This finding suggests a potential risk in relying solely on standard endotracheal tube insertion depths (e.g., 23 cm for males, 21 cm for females), which could lead to hazardous over-insertion in patients with shorter airways [15]. Such variability demands individualised approaches to airway management rather than population-based protocols.

Knowledge of total and segmental lengths is vital for tracheal resection and reconstruction planning. The amount of trachea that can be safely resected depends on the original tracheal length and the ability to mobilise remaining segments. In patients with a total length below 10 cm, resection exceeding 5 cm representing more than half the trachea requires meticulous consideration of release manoeuvres to minimise anastomotic tension [16].

Males exhibited significantly larger measurements across nearly all parameters than females, with total tracheal length differences primarily driven by longer thoracic segments. These profound gender-based differences in central airway dimensions align with previous imaging studies using 3D workstations, though our measurements appear slightly lower. Regional variations in cadaveric studies report even shorter tracheal lengths, suggesting population-based anatomical differences warrant consideration in clinical practice [16–18].

Contrary to expectations of progressive alterations, age-related tracheal changes demonstrated unexpected non-linear patterns. Total tracheal length remained stable across adult age groups, yet cervical segments showed significant elongation specifically in the 41–50 age cohort without establishing a linear age correlation. This finding supports previous observations of tracheal growth continuing until the mid-30s before stabilising, suggesting age-related changes may be segment-specific rather than uniformly distributed [17].

### Length of the main bronchi

The variable anatomy of the right mainstem bronchus and right upper lobe bronchus represents the primary factor contributing to lateral orifice misalignment in right-sided DLT (R-DLT) [14, 19]. Our study employed three distinct measurement techniques for RBL assessment, revealing significant variability in mean values depending on methodology. This variability underscores the critical importance of standardising measurement approaches, given that multiple definitions exist in previous literature, including distances from the tracheal carina to proximal or distal margins of the upper lobe bronchial orifice [19]. Liu et al. demonstrated that the carina-to-distal approach provides superior prediction accuracy for correct R-DLT placement, reducing malposition and repositioning requirements [14].

Kim’s study established that the distance from the proximal end of the endobronchial cuff to the distal edge of the ventilation slot in the Broncho-Cath DLT (Mallinckrodt Medical Ltd., Athlone, Ireland) measures 23 mm—a critical dimension that predicts R-DLT malpositioning [20]. Our study revealed a substantially higher incidence (49.50%) of RBL2 measuring less than 23 mm, with rates of 25.84% in females and 23.65% in males. This finding aligns closely with Korean (50%) and Chinese (52.3%) populations but differs markedly from Canadian patients (25%), suggesting significant ethnic and geographic anatomical variation [19, 21, 22].

The margin of safety becomes critically narrow when the right bronchial length (carina-to-proximal method) is shorter than the standard 10-mm wide Mallinckrodt right endobronchial cuff [23]. In our study, 11.92% of patients (5.36% males, 6.56% females) had an RBL1 shorter than 10 mm, and a standard R-DLT would not be feasible for these patients. This incidence parallels that reported in American patients (11%) but is lower than in Japanese populations (20%), underscoring population-specific anatomical considerations [19, 23, 24].

LBL consistently exceeded RBL length by approximately two-fold in our study, reflecting fundamental anatomical asymmetry with profound clinical consequences. This variability in main bronchial lengths directly impacts DLT selection and placement strategies [9, 25]. For L-DLT, 1.19% of patients (0.99% females, 0.19% males) had an LBL shorter than 32 mm, which would be insufficient to accommodate a standard 32 Fr L-DLT tip and cuff without risking upper lobe occlusion. While representing a smaller percentage than right-sided complications, this finding identifies a vulnerable patient population requiring specialised attention and alternative sizing strategies. To prevent upper lobe obstruction, the critical “margin of safety“(difference between bronchus length and combined cuff/tip length) must exceed 10 mm [23].

Given that Broncho-Cath DLT represent the majority of R-DLT in clinical use, the higher incidence of RBL2 < 23 mm or RBL1 < 10 mm places a significant proportion of our population at risk for R-DLT malpositioning. These findings underscore the importance of preoperative CT evaluation in determining appropriate lung isolation strategies. When the right upper lobe bronchus is located at or above the tracheal carina, or when RBL1 measures < 10 mm, alternative methods such as EZ-blockers or L-DLT should be considered instead of R-DLT. Similarly, when LBL is < 32 mm, careful consideration of tube sizing and positioning becomes paramount. In cases where R-DLT placement remains appropriate, flexible fibreoptic bronchoscopy guidance becomes essential to ensure optimal positioning and minimise complications [19].

### Diameter of the trachea and main stem bronchi

A key finding in our study is the strong correlation between the transverse diameter measured at 1 cm proximal to the carina and the proximal diameters of both main bronchi (*r* = 0.605 for the left, *r* = 0.621 for the right bronchus). This finding establishes TRANS4 as a robust predictor for bronchial dimensions—a clinically significant advancement given the difficulty in accurately measuring bronchial dimensions from chest radiographs in a substantial percentage of patients [26]. Our predictive models, incorporating TRANS4 and demographic data, offer a refined, evidence-based approach for estimating bronchial diameters that surpasses traditional methods relying solely on height, gender, or clinical experience [27, 28].

Our multi-level measurement approach from cricoid cartilage to carina provides superior morphological assessment compared to single-point studies, revealing that the narrowest laryngotracheal diameter occurs immediately below the cricoid cartilage—the critical determinant of maximum ventilation tube size [9, 29, 30].

Understanding tracheal and bronchial diameters is essential for selecting appropriately sized endotracheal and double-lumen tubes, particularly in one-lung ventilation procedures [26]. Inappropriately sized tubes can result in serious complications, including airway trauma, pressure injury, and bronchial rupture. While internal diameter determines clinical utility—airflow resistance and effective suctioning—the outer diameter is crucial for safe passage through the narrowest part of the airway, the subglottic region [31, 32]. The left main bronchus diameter, measured 2 cm below the carina, represents a critical landmark directly corresponding to the typical location of maximal cuff diameter in L-DLT. This precise anatomical correlation enables evidence-based tube sizing that optimises the delicate balance between effective lung isolation and minimising bronchial trauma, which has been a persistent challenge in thoracic anaesthesia [33]. Our detailed bronchial measurements at proximal, intermedius, and distal levels provide crucial anatomical reference points, directly addressing the clinical imperative to secure airway isolation while preventing devastating complications from oversized cuff inflation.

Although age-related tracheal changes in adults have not been reported, our study demonstrates significant age-associated distal tracheal enlargement—particularly at the carina—supporting findings from a recent Japanese study that showed a slight but consistent increase in tracheal diameter with age [29, 34, 35]. Importantly, while ethnicity shows minimal influence on tracheal dimensions compared to sex and height, with no clinically significant differences found between Chinese and Caucasian adults, current practices of routinely selecting smaller endotracheal tubes for Chinese patients based solely on ethnicity lack evidence-based support [31]. These findings emphasize that individualised airway management should prioritise patient-specific anthropometric factors rather than ethnic generalisations. This highlights the need for comprehensive normative data across diverse populations to optimise clinical outcomes.

### Angles of the main bronchi and subcarinal

Our findings revealed a significantly larger average subcarinal angle in female subjects compared to males, contradicting previous cadaveric observations [36]. This discrepancy likely arises from gender-specific patterns of pulmonary development, where in females the lungs grow more transversely than downwards before the chest wall becomes rigid. In contrast, the diaphragmatic muscle tends to develop greater strength in males. Additionally, the dynamic nature of living diaphragmatic function differs substantially from the static positioning observed in cadaveric studies [9]. Clinically, subcarinal angle widening serves as a valuable diagnostic indicator for underlying mediastinal or cardiac pathology, emphasizing its continued relevance in radiological assessment [37].

Contrary to traditional anatomical teachings in Gray’s Anatomy, our study found that 45.53% of subjects exhibited a larger right main stem bronchus angle than the left [38].This suggests that in half of the Indian population, the left main stem bronchus maintains a more vertical orientation, potentially increasing the likelihood of foreign body lodgement in the left rather than right bronchus.

### Strength

This study represents the first comprehensive effort to establish normative reference values for central airway dimensions in adults using high-resolution CT. While geographically focused on India, the findings have far-reaching global implications, as no such detailed, standardised morphometric data currently exist for any adult population. Our study utilises state-of-the-art 128-slice dual-source CT technology with advanced dose optimisation to achieve unprecedented anatomical precision—a novel combination of advanced imaging and comprehensive sampling scale that has been underexplored in the literature. While other studies examined only one or two parameters using routine CT scans performed for various clinical indications, our approach differed fundamentally. Although these prior studies required radiologically normal thoracic anatomy per inclusion criteria, they inherently relied on scans that were not completely regular reports, potentially introducing subtle selection bias despite stringent radiological criteria for normal tracheal, pulmonary, and cardiac anatomy. Most importantly, our findings directly challenge fundamental anatomical assumptions, providing population-specific evidence that could revolutionise clinical practice standards for airway management in Indian and potentially broader Asian populations.

### Limitation

Despite its comprehensive scope, this single-centre retrospective study represents a geographically and ethnically homogeneous Indian population, potentially limiting generalizability to other ethnic groups given established anatomical variations across populations and the critical need for population-specific normative data in precision medicine approaches.

## Conclusion

This study establishes the first comprehensive normative reference values for central airway dimensions in Indian adults using high-resolution CT, revealing critical anatomical variations with direct implications for patient safety in surgical and anaesthetic practice. Key findings include significant gender-based differences in airway dimensions, with males exhibiting larger tracheal and bronchial measurements, and a high prevalence of short right main bronchi (49.5% of subjects), which increases the risk of double-lumen tube misplacement. Additionally, 23.45% of participants had tracheal lengths below 10 cm, challenging standard endotracheal tube insertion protocols and underscoring the need for individualised airway management.

The strong correlation between distal tracheal diameter and bronchial dimensions (*r* > 0.6) provides a reliable tool for evidence-based device selection, reducing the risks of airway trauma, malposition, and other complications. The study also highlights the clinical importance of preoperative CT evaluation to guide lung isolation strategies, particularly in patients with anatomical variations predisposing them to complications.

These population-specific normative data emphasize the necessity of moving beyond generalised protocols to adopt precision-based approaches in airway management. By integrating these findings into clinical practice, anaesthesiologists and surgeons can enhance patient safety, optimise surgical outcomes, and minimise morbidity associated with airway interventions. Future research should focus on validating these findings in multiethnic cohorts and developing tailored guidelines for diverse populations.

**Table 3b:** Bronchial angles by age group

Parameters	Group 1 (*n* = 80)	Group 2 (*n* = 83)	Group 3 (*n* = 99)	Group 4 (*n* = 115)	Group 5 (*n* = 126)	*p* value
RBA	37.18 ± 6.96	40.41 ± 6.86	38.12 ± 7.61	39.24 ± 7.76	40.16 ± 7.68	0.012
LBA	37.37 ± 7.45	39.89 ± 6.40	38.89 ± 6.24	40.40 ± 7.21	38.87 ± 7.22	0.055
SCA	68.68 ± 19.34	78.76 ± 19.16	71.52 ± 17.40	76.54 ± 19.09	74.50 ± 19.21	0.005
RUA	114.76 ± 11.24	117.35 ± 10.75	115.28 ± 9.23	115.55 ± 11.22	117.27 ± 11.21	0.332

**Table 3c:** Bronchial dimensions by age group

Parameters	Group 1 (*n* = 80)	Group 2 (*n* = 83)	Group 3 (*n* = 99)	Group 4 (*n* = 115)	Group 5 (*n* = 126)	*p* value
RBL1	15.85 ± 4.88	15.93 ± 4.84	15.46 ± 4.94	15.00 ± 4.89	16.26 ± 5.25	0.380
RBL2	23.40 ± 4.14	23.05 ± 4.48	22.71 ± 4.07	22.84 ± 4.70	23.54 ± 4.53	0.580
RBL3	24.68 ± 4.28	25.54 ± 4.17	24.27 ± 3.66	24.92 ± 4.07	25.56 ± 3.82	0.079
P-RBD	14.24 ± 2.81	14.43 ± 2.48	14.37 ± 2.46	14.53 ± 2.55	14.91 ± 3.35	0.577
D-RBD	14.93 ± 2.73	14.86 ± 2.50	14.82 ± 2.61	14.73 ± 2.80	15.19 ± 3.04	0.792
RUBD	9.60 ± 2.79	8.81 ± 2.39	9.33 ± 2.12	9.66 ± 2.21	9.19 ± 2.37	0.111
LBL	45.11 ± 5.22	44.46 ± 6.31	46.06 ± 5.81	44.45 ± 4.84	45.88 ± 5.81	0.106
P-LBD	12.86 ± 3.10	13.27 ± 2.75	12.69 ± 2.51	13.05 ± 2.46	13.59 ± 2.76	0.121
M-LBD	10.12 ± 1.95	10.40 ± 1.82	10.44 ± 2.04	10.71 ± 1.87	11.08 ± 2.09	0.011
D-LBD	10.71 ± 2.87	10.67 ± 3.56	10.41 ± 2.86	10.72 ± 2.90	10.66 ± 3.10	0.944

Note: Values are presented as mean ± standard deviation. Statistically significant (*p* < 0.05)

**Table 4:** Multiple linear regression analysis predicting P-LBD and P-RBD from TRANS4

*P*-LBD						
	β	SE	95% CI		*p*	*R*²
			Lower	Upper		
**TRANS4**						
Crude	0.442	0.025	0.391	0.493	< 0.001	0.367
Model 1	0.414	0.027	0.359	0.469	< 0.001	0.376
**P-RBD**						
	**β**	**SE**	**95% CI**		**p**	**R²**
**TRANS4**			**Lower**	**Upper**		
Crude	0.465	0.026	0.414	0.516	< 0.001	0.386
Model 1	0.422	0.028	0.367	0.477	< 0.001	0.405

β = regression coefficient; SE = standard error; CI = confidence interval; R² = coefficient of determination; P-LBD = Proximal Left bronchus diameter, P-RBD = Proximal Right Bronchus Diameter. Crude model = unadjusted; Model 1 = adjusted for age and gender

## Data Availability

The datasets used and analysed during this study are available from the corresponding author upon reasonable request.

## Abbreviations

ANOVA: Analysis of Variance
AP: Anterior-Posterior
C-TL: Cervical Tracheal Length
CSA: Cross-Sectional Area
CT: Computed Tomography
DLT: Double-Lumen Endobronchial Tube
D-RBD: Distal Right Bronchus Diameter
D-LBD: Distal Left Bronchus Diameter
HRCT: High-Resolution Computed Tomography
I-TL: Intrathoracic Tracheal Length
LBA: Left Bronchial Angle
LBL: Left Main Bronchus Length
L-DLT: Left-Sided Double-Lumen Endobronchial Tube
M-LBD: Mid (intermedius) Left Bronchus Diameter
MPR: Multiplanar Reconstruction
P-LBD: Proximal Left Bronchus Diameter
P-RBD: Proximal Right Bronchus Diameter
RBA: Right Bronchial Angle
RBL: Right Main Bronchus Length
R-DLT: Right-Sided Double-Lumen Endobronchial Tube
RUA: Right Upper Lobe Bronchial Angle
RUBD: Right Upper Bronchus Diameter
SCA: Subcarinal Angle
TL: Total Tracheal Length
TRANS: Transverse

## References

[1] Brown RH, Henderson RJ, Sugar EA, Holbrook JT, Wise RA, Brown RH. Reproducibility of airway luminal size in asthma measured by HRCT. J Appl Physiol [Internet]. 2017;123:876–83. Available from: http://www10.1152/japplphysiol.00307.2017 10.1152/japplphysiol.00307.2017 PMC5668456 28705995

[2] Hannallah M, Benumof JL, Silverman PM, Kelly LC, Lea D. Evaluation of an approach to choosing a left double-lumen tube size based on chest computed tomographic scan measurement of left mainstem bronchial diameter. J Cardiothorac Vasc Anesth [Internet]. 1997;11(2):168–71. Available from: 10.1016/S1053-0770(97)90208-1 10.1016/s1053-0770(97)90208-1 9105987

[3] Chow MYH, Liam BL, Thng CH, Chong BK. Predicting the size of a double-lumen endobronchial tube using computed tomographic scan measurements of the left main bronchus diameter. Anesth Analg [Internet]. 1999;88(2). Available from: https://journals.lww.com/anesthesia-analgesia/fulltext/1999/02000/predicting_the_size_of_a_double_lumen.14.aspx.10.1097/00000539-199902000-00014 10.1097/00000539-199902000-00014 9972745

[4] Hautmann H, Gamarra F, Henke M, Diehm S, Huber RM. High frequency jet ventilation in interventional fiberoptic bronchoscopy. Anesth Analg [Internet]. 2000;90(6).Available from: https://journals.lww.com/anesthesia-analgesia/fulltext/2000/06000/high_frequency_jet_ventilation_in_interventional.34.aspx.10.1097/00000539-200006000-00034 10.1097/00000539-200006000-00034 10825336

[5] Singh-Radcliff N. 5-Minute anesthesia consult. Lippincott Williams & Wilkins; 2012.

[6] Hillel AT, Karatayli-Ozgursoy S, Samad I, Best SRA, Pandian V, Giraldez L, et al. Predictors of posterior glottic stenosis: A multi-institutional case-control study. Annals Otology Rhinology Laryngology. 2016;125(3):257–63. 10.1177/0003489415608867. 10.1177/0003489415608867 PMC4742396 26466860

[7] Mahajan S, Mahajan A, Lalit M, Verma P. Morphometry of adult human trachea and its clinical implications: A cadaveric study in Northern india. J Clin Diagn Res. 2022;16(7). 10.7860/JCDR/2022/55145.16574

[8] Kim IS, Song CH. The morphometric study of main bronchus in Korean cadaver. Korean J Phys Anthropol. 2017;30(1):7. 10.11637/aba.2017.30.1.7.

[9] Mi W, Zhang C, Wang H, Cao J, Li C, Yang L, et al. Measurement and analysis of the tracheobronchial tree in Chinese population using computed tomography. PLoS ONE. 2015;10(4). 10.1371/journal.pone.0123177. 10.1371/journal.pone.0123177 PMC4404098 25894917

[10] Szelloe P, Weiss M, Schraner T, Dave MH. Lower airway dimensions in pediatric patients—A computed tomography study. Paediatr Anaesth. 2017;27(10):1043–9. 10.1111/pan.13210. 28846178 10.1111/pan.13210

[11] Chalwadi UK, Swamy N, Agarwal A, Gauss CH, Greenberg SB, Lyons KA. Determining normal values for lower trachea and bronchi size in children by computed tomography (CT). Pediatr Pulmonol. 2021;56(9):2940–8. 10.1002/ppul.25536. 34133085 10.1002/ppul.25536

[12] Kuo W, Ciet P, Andrinopoulou ER, Chen Y, Pullens B, Garcia-Peña P, et al. Reference values for central airway dimensions on CT images of children and adolescents. Am J Roentgenol. 2018;210(2):423–30. 10.2214/AJR.17.18597. 29261353 10.2214/AJR.17.18597

[13] Premakumar Y, Griffin MF, Szarko M. Morphometric characterisation of human tracheas: focus on cartilaginous ring variation. BMC Res Notes. 2018;11(1). 10.1186/s13104-018-3123-1. 10.1186/s13104-018-3123-1 PMC5771073 29338790

[14] Liu Z, Liu M, Zhao L, Qi X, yu Y, Liang S, et al. Comparison of the accuracy of three methods measured the length of the right main stem bronchus by chest computed tomography as a guide to the use of right sided double-lumen tube. BMC Anesthesiol. 2022;22(1). 10.1186/s12871-022-01744-z. 10.1186/s12871-022-01744-z PMC9387006 35982403

[15] Varshney M, Sharma K, Kumar R, Varshney PG. Appropriate depth of placement of oral endotracheal tube and its possible determinants in Indian adult patients. Indian J Anaesth. 2011;55(5):488–93. 10.4103/0019-5049.89880. 22174466 10.4103/0019-5049.89880 PMC3237149

[16] Matsuoka S, Shimizu K, Koike S, Takeda T, Miura K, Eguchi T, et al. Significance of the evaluation of tracheal length using a three-dimensional imaging workstation. J Thorac Dis. 2022;14(11):4276–84. 10.21037/jtd-22-595. 36524079 10.21037/jtd-22-595 PMC9745505

[17] Jit H, Jit I. Dimensions & shape of the trachea in the neonates, children & adults in Northwest India. Indian J Med Res. 2000;112:27. 11006658

[18] Datta DD, Kundu DD, ProfDrS P, Das DrA. A morphometric study of adult human trachea in West Bengal population. Int J Med Res Rev. 2019;7(1):36–42. 10.17511/ijmrr.2019.i01.07.

[19] Chen Y, Guo Y, Mi W, Zhang C, Wang H, Zhao D, et al. Anatomy of the right upper lobe revisited and clinical considerations in Chinese population. PLoS ONE. 2020;15(11 November). 10.1371/journal.pone.0242178. 10.1371/journal.pone.0242178 PMC7688111 33237948

[20] Kim JH, Park SH, Han SH, Nahm FS, Jung CK, Kim KM. The distance between the carina and the distal margin of the right upper lobe orifice measured by computerised tomography as a guide to right-sided double‐lumen endobronchial tube use. Anaesthesia. 2013;68(7):700–5. 10.1111/anae.12208. 23656604 10.1111/anae.12208

[21] Bussières JS, Gingras M, Perron L, Somma J, Frenette M, Couture EJ, et al. Right upper lobe anatomy revisited: a computed tomography scan study. Can J Anesth. 2019;66(7):813–9. 10.1007/s12630-019-01342-7. 30838521 10.1007/s12630-019-01342-7

[22] Wong DT, Kumar A. Case report: endotracheal tube malposition in a patient with a tracheal bronchus. Can J Anesth. 2006;53(8):810. 10.1007/BF03022798. 16873348 10.1007/BF03022798

[23] Benumof JL, Partridge BL, Salvatierra C, Keating J. Margin of safety in positioning modern double-lumen endotracheal tubes. Anesthesiology. 1987;67(5):729–38. 10.1097/00000542-198711000-00018. 3674473 10.1097/00000542-198711000-00018

[24] Hagihira S, Mashimo T, Takashina M. Application of a newly designed right-sided, double-lumen endobronchial tube in patients with a very short right mainstem bronchus. Anesthesiology. 2008;109:565–8. 10.1097/ALN.0b013e31818344bd. 18719454 10.1097/ALN.0b013e31818344bd

[25] Kim D, Son JS, Ko S, Jeong W, Lim H. Measurements of the length and diameter of main bronchi on three-dimensional images in Asian adult patients in comparison with the height of patients. J Cardiothorac Vasc Anesth. 2014;28(4):890–5. 10.1053/j.jvca.2013.05.029. 24103712 10.1053/j.jvca.2013.05.029

[26] Shah SB, Hariharan U, Chawla R. Choosing the correct-sized adult double-lumen tube: quest for the holy Grail. Annals of Cardiac Anaesthesia. 2023;26:124–32. 10.4103/aca.aca_140_22 10.4103/aca.aca_140_22 PMC10284481 37706375

[27] Pedoto A. How to choose the Double-Lumen tube size and side. The eternal debate. Anesthesiol Clin. 2012;30:671–81. 10.1016/j.anclin.2012.08.001. 23089502 10.1016/j.anclin.2012.08.001

[28] Slinger P. Choosing the appropriate double-lumen tube: a glimmer of science comes to a dark Art. J Cardiothorac Vasc Anesth. 1995;9(2):117–8. 10.1016/S1053-0770(05)80179-X. 7780064 10.1016/S1053-0770(05)80179-X

[29] Coordes A, Rademacher G, Knopke S, Todt I, Ernst A, Estel B, et al. Selection and placement of oral ventilation tubes based on tracheal morphometry. Laryngoscope. 2011;121(6):1225–30. 10.1002/lary.21752. 21557233 10.1002/lary.21752

[30] Ulusoy M, Uysal II, Kivrak AS, Ozbek S, Karabulut AK, Paksoy Y et al. Age and gender related changes in bronchial tree: a morphometric study with multidedector CT. Eur Rev Med Pharmacol Sci. 2016;20(16). 27608892

[31] Tai A, Corke C, Joynt GM, Griffith J, Lunn D, Tong PWY. A comparative study of tracheal diameter in Caucasian and Chinese patients. Anaesth Intensive Care. 2016;44. 10.1177/0310057X1604400603. 10.1177/0310057X1604400603 27832558

[32] Karmali S, Rose P. Tracheal tube size in adults undergoing elective surgery – a narrative review. Anaesthesia. 2020;75:1529–39. 10.1111/anae.15041 10.1111/anae.15041 32415788

[33] Lee JW, Son JS, Choi JW, Han YJ, Lee JR. The comparison of the lengths and diameters of main bronchi measured from two-dimensional and three-dimensional images in the same patients. Korean J Anesthesiol. 2014;66(3):189–94. 10.4097/kjae.2014.66.3.189. 24729839 10.4097/kjae.2014.66.3.189 PMC3983413

[34] Sakai H, Nakano Y, Muro S, Hirai T, Takubo Y, Oku Y, et al. Age-related changes in the trachea in healthy adults. Oxygen transport to tissue XXXI. Springer; 2010. pp. 115–20. 10.1007/978-1-4419-1241-1_16. 10.1007/978-1-4419-1241-1_16 20204780

[35] Brodsky JB, Macario A, Mark JBD. Tracheal diameter predicts Double-lumen tube size: A method for selecting left double-lumen tubes. 10.1097/00000539-199604000-00032 8615510

[36] Shrestha A, Ranjit N, Bhandari R, Adhikari B, Gautam J. Measurement of subcarinal angle: A cadaveric study. J Inst Med Nepal. 2019;41(1):20–3. 10.3126/jiom.v41i1.28587.

[37] Karabulut N. CT assessment of tracheal carinal angle and its determinants. Br J Radiol. 2005;78(933):787–90. 10.1259/bjr/75107416. 16110098 10.1259/bjr/75107416

[38] Standring S. Gray’s anatomy: the anatomical basis of clinical practice, expert consult. Aubrey Durkin; 2009.

